# Supplementary material for: An electron-deficient carbon current collector for anode-free Li-metal batteries
Source: Nat Commun. 2021 Sep 20;12:5537. doi: 10.1038/s41467-021-25848-1 (PMC8452779; doi:10.1038/s41467-021-25848-1)
Supplement: Supplementary file 1 — Supplementary Informations [file 41467_2021_25848_MOESM1_ESM.pdf]

Supplementary Information

An electron-deficient carbon current collector for anode-free Li metal batteries

*Hyeokjin-Kwon[1], Ju-Hyuk Lee[1], Youngil Roh[1], Jaewon Baek[1], Dong Jae Shin[1], Jong Keon Yoon[2], Hoe Jin Ha[2], Je Young Kim[2], Hee-Tak Kim[1,3]**

[1]Department of Chemical and Biomolecular Engineering, KAIST, 291 Daehak-ro, Yuseong-gu, Daejeon 34141, Republic of Korea

[2]Battery R&D, LG Chem, 188, Munji-ro, Yuseong-gu, Daejeon, 34122, Republic of Korea

[3]Advanced Battery Center, KAIST Institute for the NanoCentury, KAIST, 291, Daehak-ro, Yuseong-gu, Daejeon 34141, Republic of Korea

*Corresponding author: Email: heetak.kim@kaist.ac.kr; Tel.: +82-42-350-3916; Fax: +82-42-350-3910

Supplementary note 1, DFT calculation

All density functional theory (DFT) calculations were performed with the program package DMol[3] in Materials Studio from Accerlrys, Inc. on personal computers. DMol[3] uses numerical orbitals as basis functions, each of which corresponds to atomic orbitals. These works utilize a double-numeric-plus-polarization (DNP) function and a global orbital cutoff of 5.4 Å. The size of the DNP basis set is comparable to Gaussian 6-31G(d), but the DNP is more accurate than the corresponding Gaussian basis set[1]. DFT calculations were carried out with a gradient-corrected (GGA) functional with the Perdew-Bueke-Ernzerhof (PBE) exchange correlation functional. Tolerances of energy, gradient, and displacement convergence were 0.00001 hartree, 0.002 hartree/Å, and 0.005 Å, respectively. To avoid the interaction caused by the periodicity, a vacuum layer of 20 Å along the z-direction was used for calculations. The slab models were all built on the basis of a monolayer graphite (001) surface in a $3 \times 3 \times 1$ supercell. By doing so, the Dirac points (K point) of the 1 x 1 grapehene BZ are folded into the Γ point of the 3 x 3 supercell[2], and $3 \times 3 \times 1$ k-point Monkhorst-Pack grids were adopted to the Brillouin zone. Band structures of lithiated MV structures were mapped onto the K point of the folded Brillouin zone. The force tolerance of self-consistent-field (SCF) cycles was 1.0×10^{-6}. Grimme's DFT-D2 method was adopted to account for the Van der Waals interactions, and this method was optimized for several DFT functionals. The adsorption free energies (ΔG_{ads}) of Li on different carbon surfaces were calculated as follows:

$$\Delta G_{ads} = \{E_{BLG+Li_z} - [E_{BLG} - zE_{Li}]\}/z \qquad (1)$$

where E_{BLG+Li_z}, E_{BLG}, and E_{Li} are the total energies of bilayer graphene (BLG) after z number of Li adsorption, the pristine BLG surface, and isolated Li atoms, respectively; z is the number of adsorbed Li atoms.

The average potential of lithiated materials can be calculated by Eqn. (2).

$$V(z_2) - V(z_1) = -\frac{G_{BLG+z_2Li} - G_{BLG+z_1Li} - (z_2 - z_1)G_{Li}}{(z_2 - z_1)e} \qquad (2)$$

where $V(z_2) - V(z_1)$ means the potential change with the increment of the number of Li atom from z_1 to z_2; G_{BLG+z_2Li}, G_{BLG+z_1Li}, and G_{Li} are Gibbs free energy of BLG with z_2 Li atoms, BLG with z_1 Li atom, and isolated lithium atom, respectively; and e is the elemental charge of an electron.

Supplementary note 2, Electrochemical analysis of Li nucleation mechanism

For a quantitative analysis of Li nucleation, chronoamperometry (CA) was performed at several potentials and the results were interpreted with Scharifker and Hills' model. Test cells were assembled with a 16 pi Li counter electrode, 16 pi carbon paper or Cu current collector, 19 pi Celgard separator ,and 100 μl of 1M $LiPF_6$ EC/DEC+10% FEC+1% VC electrolyte. To minimize the currents from SEI layer formation and electrochemical lithiation of graphitic carbon, p-CP and d-CP were fully lithiated by decreasing the potential to 0.01 V and holding the potential at 0.01 V with a current cut-off of 10 μA before conducting galvanostatic CA at 0.5 mA cm^{-2}. Non-dimensional current-time transient curves ($(I/I_m)^2$ vs. (t/t_m)) for p-CP and d-CP were fitted with instantaneous nucleation or the progressive nucleation model. The non-dimensional current-time responses for instantaneous nucleation and progressive nucleation are given in Eqns. (3) and (4), respectively:

$$(\frac{I}{I_m})^2 = 1.9542\frac{[1-\exp\left(-1.2564\left(\frac{t}{t_m}\right)\right)]^2}{(\frac{t}{t_m})} \quad (3)$$

$$(\frac{I}{I_m})^2 = 1.2254\frac{[1-\exp\left(-2.3367\left(\frac{t}{t_m}\right)^2\right)]^2}{(\frac{t}{t_m})} \quad (4)$$

Nuclei number densities of Li (N_0) were calculated from the CA data based on Scharifker and Hills' instantaneous nucleation-growth model by using the following equations:

$$I_m{}^2 t_m = 0.1629(nFAC_i)^2 D_i \quad (5)$$

$$N_0 = \frac{I_m{}^2}{0.41(nFAC_iD_i)^2 k_i} \quad (6)$$

$$k_i = \frac{4}{3}(\frac{8\pi C_i M}{\rho})^{1/2} \quad (7)$$

where n is the number of transferred electrons participating in the electrochemical reaction, F is the Faraday constant (96485 C mol^{-1}), A is the effective electroactive surface area, C_i is the Li^+ concentration of electrolyte, D_i is the Li^+ diffusion coefficient, M is the atomic weight of a Li atom (6.94 g mol^{-1}), and ρ is the density of Li metal (0.534 g cm^{-3}). The current-time transient curves were obtained using a VSP Potentiostat System (Bio-logic).

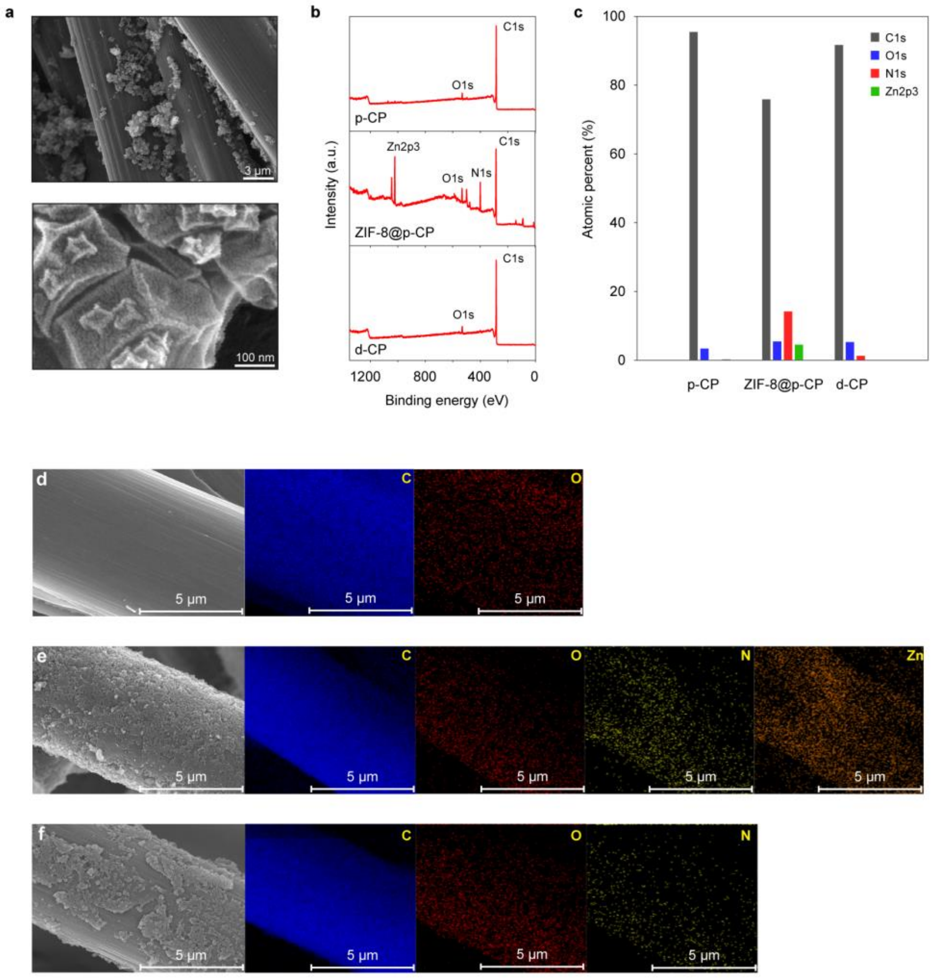

Supplementary Figure 1. Morphological and elemental analysis of the current collectors. a) SEM images of ZIF-8@p-CP. The nucleation of ZIF-8 crystal on p-CP fiber was triggered by a high binding energy between Zn^{2+} and surface carbon atoms of p-CP[3]. **b)** XPS survey spectra of p-CP, ZIF-8@p-CP, and d-CP. **c)** Atomic percentages from the XPS survey spectra for p-CP, ZIF-8@p-CP, and d-CP. **d-f)** FE-SEM image and EDX mappings: d) p-CP, e) ZIF-8@p-CP, and f) d-CP.

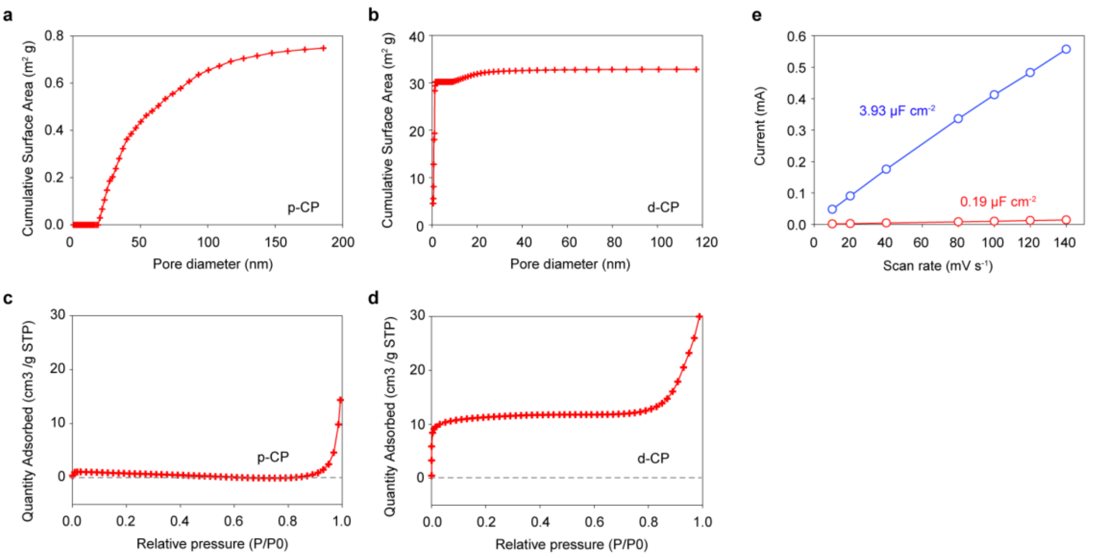

Supplementary Figure 2. Surface area measurement for p-CP and d-CP electrode. a, b) BJH adsorption cumulative surface area for a) p-CP and b) d-CP. **c, d)** The change of the amount adsorbed on c) p-CP and d) d-CP with increasing relative pressure. **e)** Plot of capacitance current as a function of scan rate for p-CP (red) and d-CP (blue).

The surface area of d-CP excluding the contribution of micro and mesopores (< 50 nm) was 0.19 $m^2 g^{-1}$, which is similar to that of p-CP (0.17 $m^2 g^{-1}$) (Supplementary Figs. 2a, b). This means that the coating of carbonized ZIF-8 does not change the bulk morphology of the pristine carbon paper current collector, while only introducing defects on the surface basal plane. The total surface area of d-CP including the contribution of micro and mesopores was as large as 32.9 $m^2 g^{-1}$. This is about 44 times larger than that of p-CP due to its hole opening structure[4,5], which is a similar result with the double layer capacitance measurement (d-CP has 20.7 times larger capacitance than p-CP). Since micropores (< 2 nm) are hardly involved in electrolyte decomposition due to the larger carbonate electrolyte molecules[6-8], the BET surface area excluding the contribution from micropores (0.74 for p-CP and 2.64 $m^2 g^{-1}$ for d-CP) is regarded as the electrochemically active surface area for the LSV analysis (Fig. 3b). In spite of its large surface area, d-CP showed suppressed electrolyte decomposition, as indicated by the higher initial CEs (Fig. 3a). By multiplying the BET surface area by the apparent weight of current collectors ($5.5x10^{-3}$ g cm^{-2} for p-CP and $3.9x10^{-3}$ g cm^{-2} for d-CP), we could obtain the apparent electrochemically active surface area of each current collector (40.7 cm^{-2} for 1 cm^2-sized p-CP and 103.0 cm^2 for 1 cm^2-sized d-CP electrode).

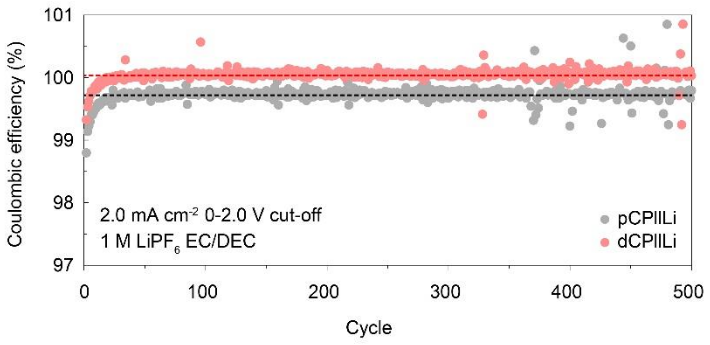

Supplementary Figure 3. Coulombic efficiency test of carbon current collector||Li half cell. The red and black dotted lines indicate average coulombic efficiencies of p-CP and d-CP half cells.

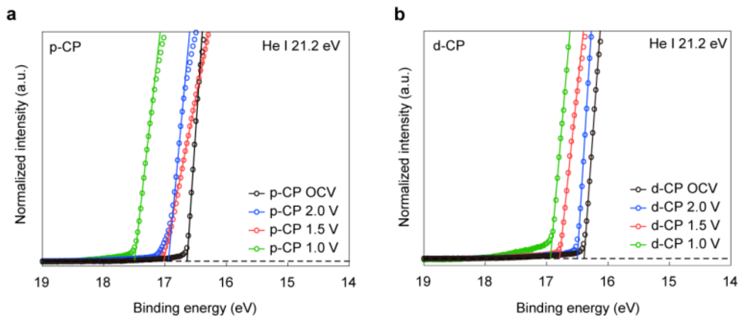

Supplementary Figure 4. UV-ray photoelectron spectroscopy (UPS) analysis. a, b) Secondary cut-off region in ex-situ UPS plot along with descending potential (from open circuit voltage (~3.0 V) to 1.0 V vs. Li reference electrode) for a) p-CP and b) d-CP. Electrochemical lithiation for the UPS analysis was conducted with 1 M $LiPF_6$ EC/DEC electrolyte at 50 μA cm^{-2}. In this experiment, FEC was removed from the electrolyte because the SEI formed from FEC can screen the signals from the lithiated carbon surfaces. The cut-off voltage for lithiation was varied as 2.0, 1.5, and 1.0 V.

From the cut-off binding energies in the UPS signals (Supplementary Figs. 4a and b), the work functions of the p-CP and d-CP surface at the various potentials were determined. The increased cut-off binding energy with lithiation means a decrease in work function and an increase in the Fermi level with lithiation for both p-CP and d-CP.

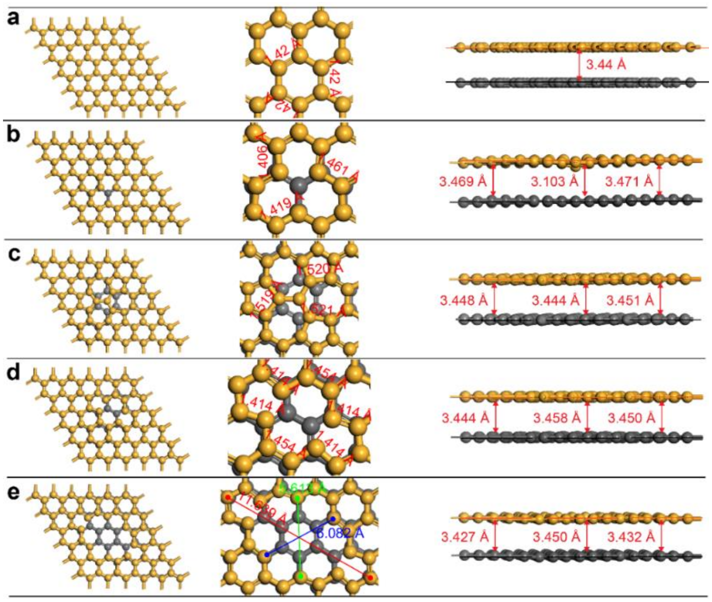

Supplementary Figure 5. Optimized atomic structure of perfect BLG and various vacancy defective BLGs. a) perfect BLG, **b)** SV, **c)** DV555-777, **d)** DV585, and **e)** MV.

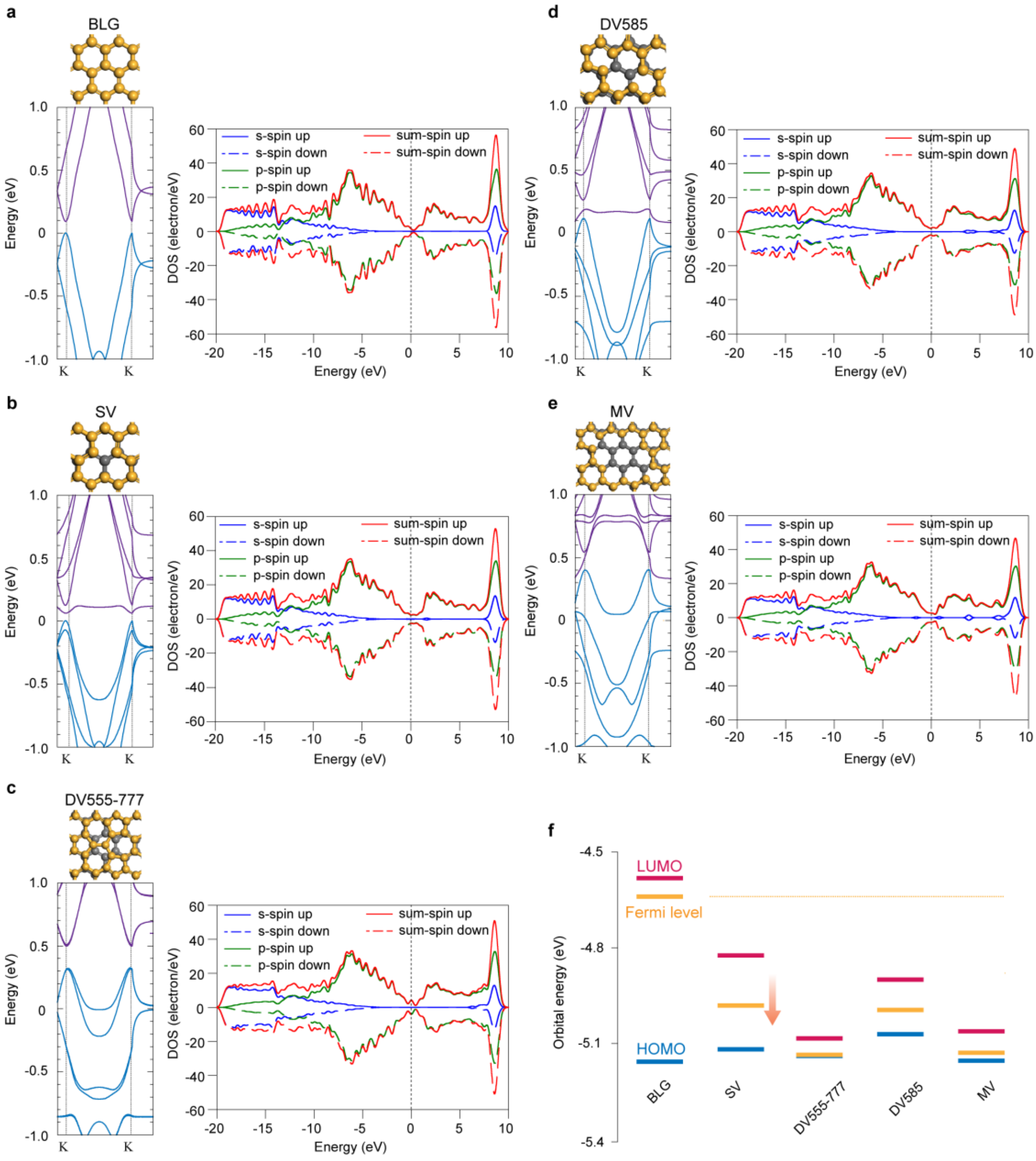

Supplementary Figure 6. Electronic structure and partial density of states (pDOS) of perfect BLG and various vacancy defect-containing BLG. a) Perfect BLG, **b)** SV, **c)** DV555-777, **d)** DV585, and **e)** MV. Fermi level was set to zero. **f)** Comparison of HOMO, LUMO, and Fermi level for the perfect bilayer graphene and various defective BLGs. LUMO, HOMO, and Fermi level are indicated as red, blue, and yellow lines, respectively.

From the higher DOSs at the Fermi level due to P_z states (Supplementary Fig. 6a-e) and lower band gap energies (Supplementary Fig. 6f) for the defect structures in comparison with BLG, it can be expected that these defective structures are electronically conductive.

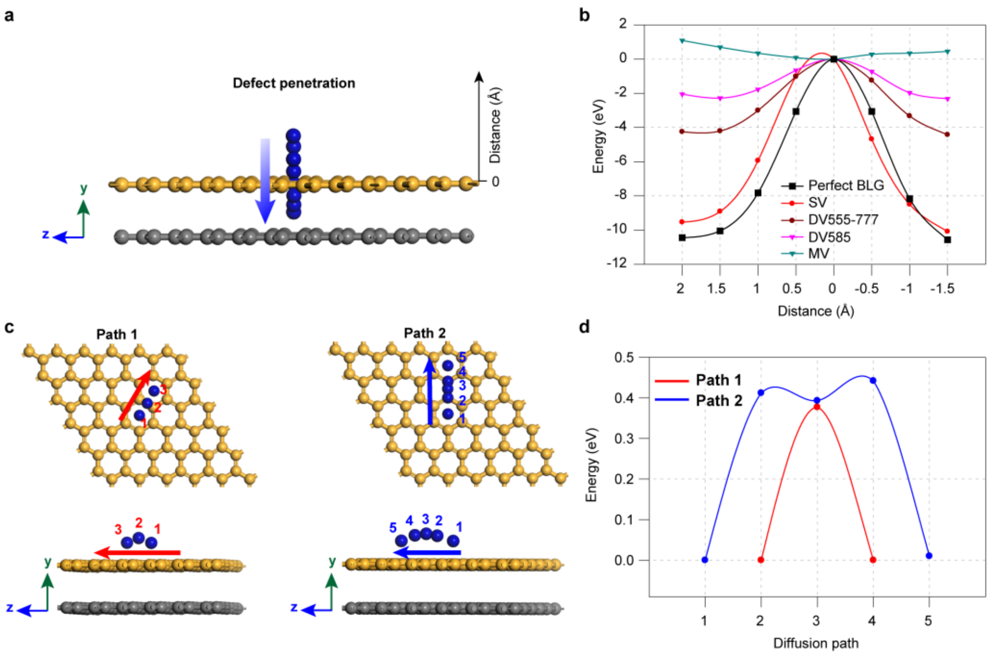

Supplementary Figure 7. Li ion diffusion across the bilayer and along in-plane direction of BLG. a) Trajectory of Li ion movement across BLG structure. **b)** Energy barrier at various positions for the Li ion moving across BLG structure, as described in a). The position of the upper graphene layer set to zero distance. **c)** Trajectories of in-plane Li ion movement across C-C bond and along C-C bond on perfect BLG. **d)** Energy barrier at various positions for the Li ion moving along perfect BLG surface (Path 1: across C-C bond, Path 2: along C-C bond). The diffusion path numbers are indicated in b).

For perfect BLG and SV structures, Li atom has difficulty penetrating though the planes, and therefore it adsorbs on the surfaces instead. The energy barrier for Li ion diffusion along the through-plane direction (Supplementary Fig. 7a) is obviously decreased with increasing vacancy size (Perfect BLG: 10.2 eV, SV: 9.7 eV, DV555-777: 4.1 eV, DV585: 2.1 eV) (Supplementary Fig. 7b). Contrary to single and double vacancy defect-containing BLGs, the MV-containing BLG does not require an activated process for Li penetration through the MV due to the absence of an energy barrier across the BLG structure. In comparison with the energy barrier for the in-plane diffusion on perfect BLG (Supplementary Fig. 7c, d), the energy barrier for through-plane diffusion across the MV-containing BLG is very small, offering a scenario of Li lithiation into d-CP rather than adsorption on the surface.

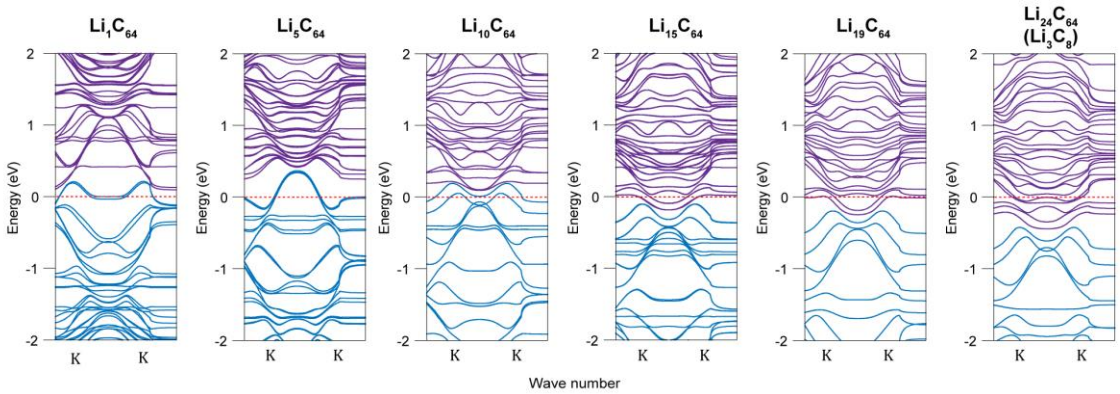

Supplementary Figure 8. Band structures of lithiated MV structures with different lithium content from Li_1C_{64} to $Li_{24}C_{64}$ (Li_3C_8).

With an increasing number of Li atoms underneath the MV, the Fermi level moves upward due to the extra valence electrons of Li atoms, which fill the part of the valence band of the MV, similar to n-type doping.

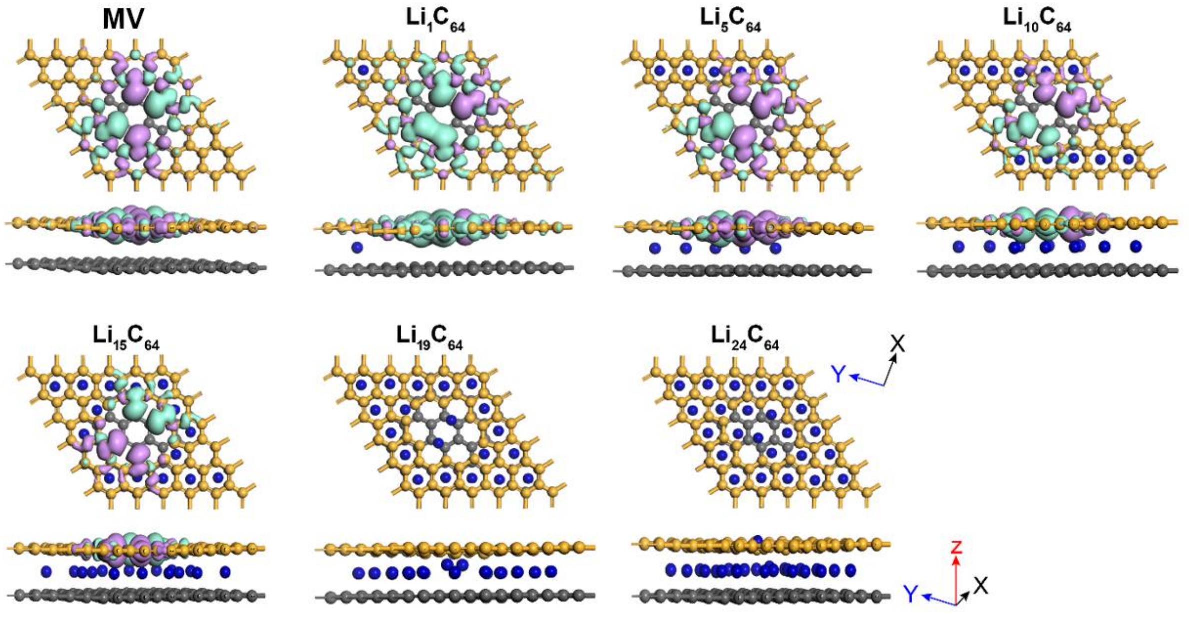

Supplementary Figure 9. The distribution of spin density with the iso-surface value of 0.005 eV/Å for lithiated MV structures. Carbon atoms in the upper layer and lower layer are represented in orange and gray, respectively. Li atoms are represented as blue spheres.

The spin-polarization of the MV structure starts to decrease at $Li_{19}C_{64}$ (Supplementary Fig. 9) upon lithiation and completely disappear sat Li_3C_8. This can be understood as the spin asymmetry and the strong magnetic momentum originating from the dangling bonds are stabilized by receiving electrons from the lithium atoms.

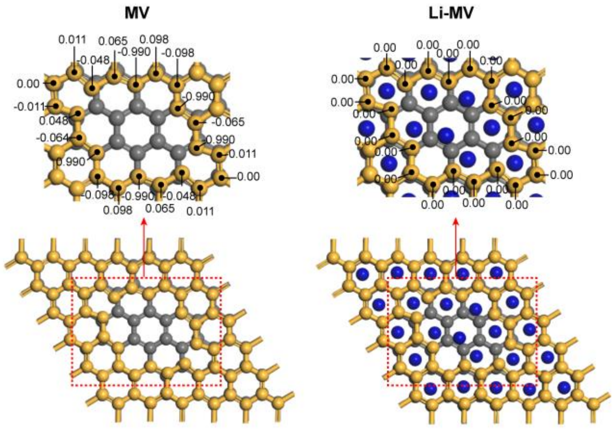

Supplementary Figure 10. Local magnetic moments (μ_B) for MV and fully-lithiated MV structure (Li_3C_8).

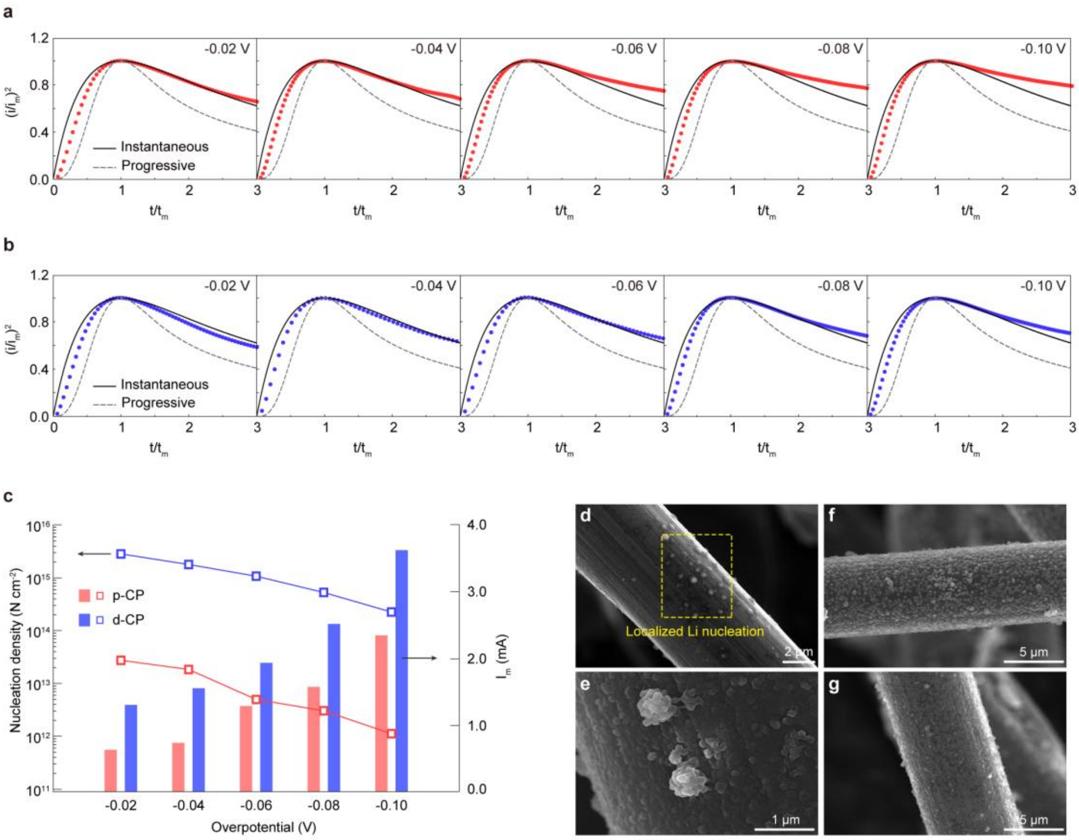

Supplementary Figure 11. Li nuclei density for p-CP and d-CP. a, b) Non-dimensional plots of $(I/I_m)^2$ as a function of t/t_m for the current density-time transients at different electrode potentials (from -0.02 to -0.10 V with an interval of 0.02 V) with a) p-CP and b) d-CP. Solid line and dashed line indicate the theoretical responses based on the instantaneous nucleation-growth model and progressive nucleation-growth model, respectively. **c)** Comparisons of nuclei number density and maximum potentiostatic current (I_m) between p-CP and d-CP. The nuclei number density was calculated based on Scharifker and Hills' instantaneous nucleation-growth model (Supplementary note 2). **d-g)** SEM images with different magnifications of d, e) p-CP and f, g) d-CP at a Li deposition capacity of d, f) 1, and e, g) 5 μAh cm^{-2}.

Regardless of the applied potential, the experimental data better fit the three-dimensional instantaneous nucleation growth mechanism. The deviations of the experimental data from the theoretical lines at $t/t_m > 1$ are attributed to a finite diffusion layer thickness that differs from the assumption of infinite diffusion layer thickness for the S-H nucleation theory[9], and to the parasitic liquid electrolyte decomposition on the Li particles[10]. In line with the SEM results (Fig. 5a-f) showing a larger number of Li nuclei on d-CP than on p-CP, the current-time transients analysis confirms that the MV structure on the defective layer promotes the nucleation of metallic Li.

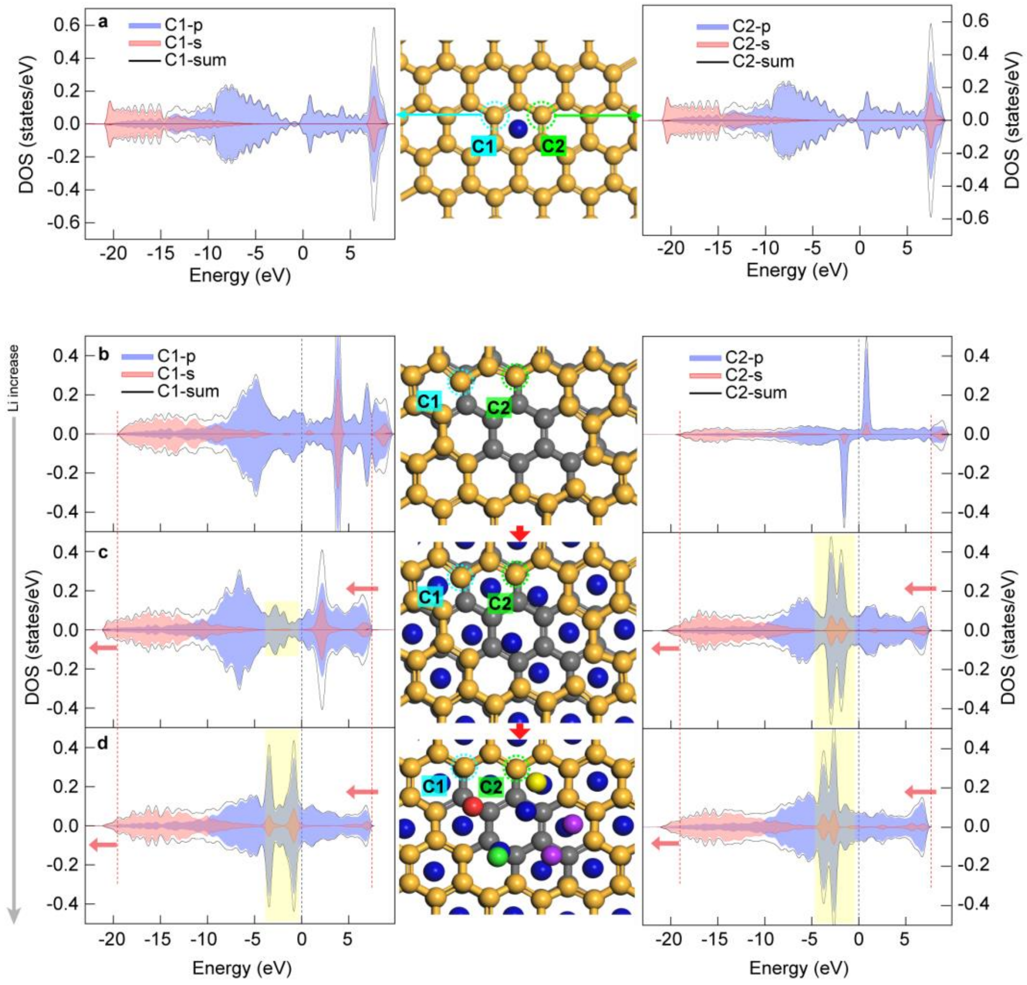

Supplementary Figure 12. pDOS analysis for the Li adsorption on perfect BLG, MV, and Li-MV. pDOS of **a)** perfect BLG with single adsorbed Li atom, **b)** MV, **c)** Li-MV (Li_3C_8), and **d)** Li-MV with five adsorbed Li atoms.

The pDOSs of two carbons (C1 and C2) in perfect BLG do not differ (Supplementary Fig. 12a). For the MV structure, as shown in Supplementary Fig. 12b, the pDOS of C2 atom shows asymmetry between up-spin and down-spin, in line with the higher magnetic momentum (0.99 μB for C2 and 0.048 μB for C1, Supplementary Fig. 10). The lithium atoms inserted into the MV structure stabilize the magnetic momentum, as shown in Supplementary Fig. 12c. The spin asymmetry of the C2 atom disappears, and the entire DOS shifts to the left (indicated by the red arrows) because of the electron transfer from the inserted lithium atoms. In the case of Li adsorption on the Li-MV (Supplementary Fig. 12d), DOS peaks in the range of 0 to -5 eV (yellow boxes) moved further to the left (indicated by the red arrows), indicating that the Li-MV receives electrons from the adsorbed Li.

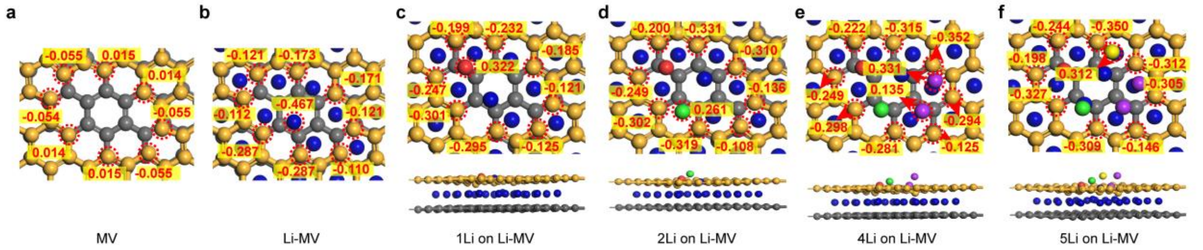

Supplementary Figure 13. The Mulliken charge distribution before and after adsorption of Li atoms on the MV and fully lithiated MV (Li_3C_8). a) MV, **b)** fully lithiated MV structure (Li_3C_8), **c)** Li_3C_8 structure with one Li atom (red sphere), **d)** one Li atom (green sphere)-added structure (c), **e)** two Li atoms (purple sphere)-added structure (d), and **f)** one Li atom (yellow sphere)-added the structure (e)[11-13]. The number of adsorbed Li is 1, 2, 4, and 5 for (c), (d), (e), and (f), respectively.

When examining the Mulliken charge distribution for the Li-MV surface with lithium adatoms on its surface, it was observed that the adsorbed lithium atoms had a partial positive charge and the carbon atoms at the edge of the MV defect structure a partial negative charge. The partial charges indicate electron transfer from lithium to the electron-deficient carbon defect structure and orbital sharing between the lithium adatom and the MV defect structure.

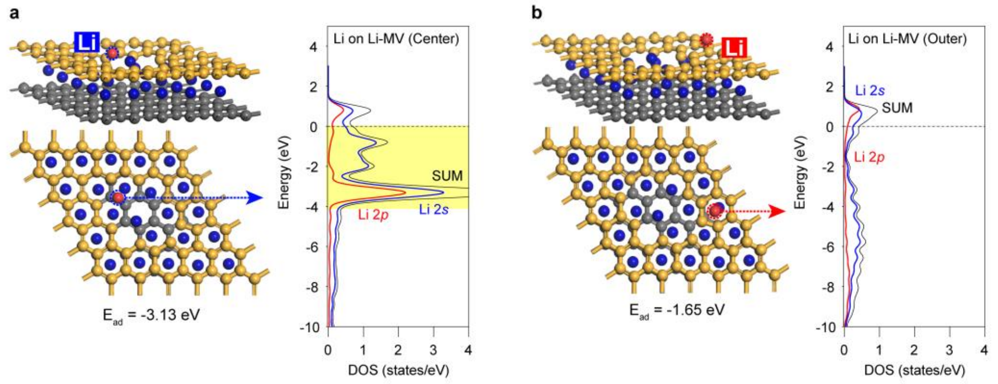

Supplementary Figure 14. Comparison of two different Li adsorption positions on Li-MV. a, b) Relaxed structures and pDOS of Li atom adsorbed on a) center and b) outer of Li-MV

Lithium binding energy is larger and the DOS peak stronger at the center of the defect than at the outer area of the defect. This is because the adsorbed lithium atom is trapped within the MV structure by sharing its electrons to nearby carbon atoms. Due to the strong binding energy, surface diffusion of Li adatom can be suppressed on the Li-MV surface.

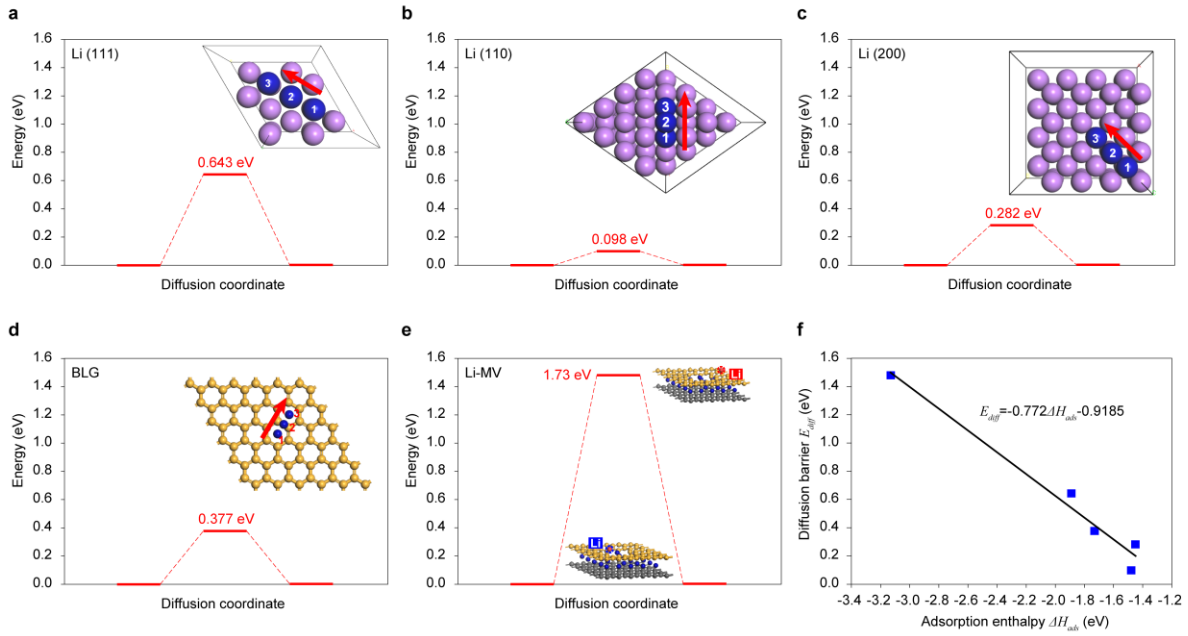

Supplementary Figure 15. Diffusional behavior of Li adatom on various substrates. a-e, Calculated Li adatom surface diffusion activation energies on (a) Li(111), (b) Li(110), (c) Li(200), (d) BLG, and (e) Li-MV. The blue atoms denote diffusing Li adatom. **f,** Brønsted−Evans−Polanyi (BEP) relation[14] between calculated adsorption enthalpy and diffusion activation energy of Li adatom on various substrate.

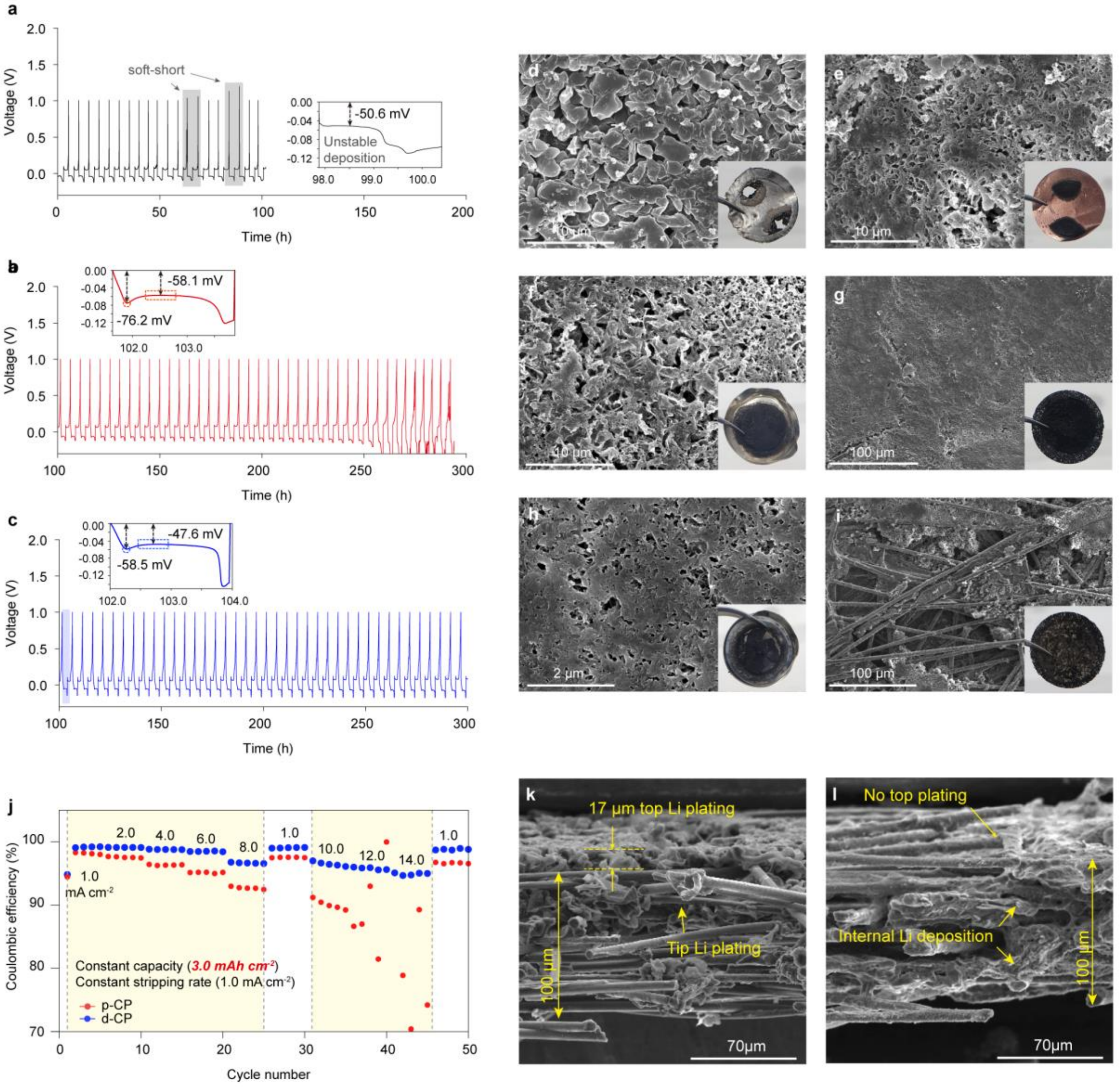

Supplementary Figure 16. Half-cell analysis of current collectors. a-c) Voltage profiles of a) the Li||Cu, b) the Li||p-CP, and c) Li||d-CP half-cell during the cycling at a current density of 2.0 mA cm^{-2}. The nucleation overpotential and plating overpotential are indicated in the insets. **d-i)** Post-mortem analysis after the half-cell cycling; d) Li electrode and e) Cu current collector of Li||Cu cell; f) Li electrode and g) p-CP of Li||p-CP cell; h) Li electrode and i) d-CP of Li||d-CP cell. **j)** Rate capabilities of the half-cells. The plating rate on current collector was varied from 1.0 to 14 mA cm^{-2}, while the stripping rate was fixed at 1.0 mA cm^{-2}. The five cycles at 1.0 mA cm^{-2} was inserted after cycling at 8.0 and 14.0 mA cm^{-2} to assess the CE recovery. **k, l)** Cross-sectional SEM images of morphology of k) p-CP and l) d-CP after Li plating at 1 mA cm^{-2} and 9 mAh cm^{-2} Li plating. For d-CP, the electrode thickness after the Li plating of 9 mAh cm^{-2} was identical to that of pristine carbon paper (100 μm) because d-CP exhibited no top Li plating.

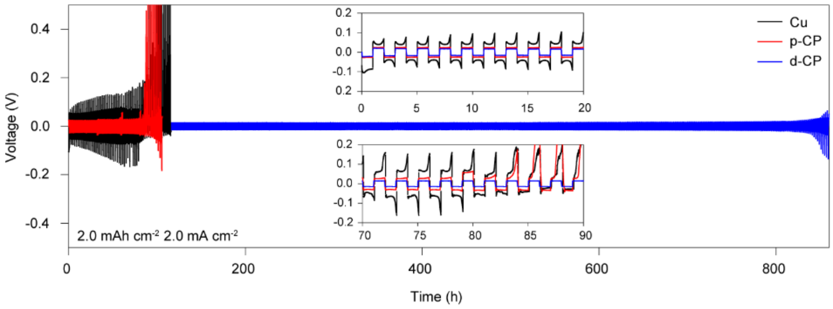

Supplementary Figure 17. Symmetric cell test with Li deposited current collectors. Voltage profiles during the galvanostatic Li plating/stripping at 2 mA cm^{-2} and 2 mAh cm^{-2} for the symmetric cells with metallic Li-deposited Cu, p-CP, and d-CP. The operation condition corresponds to 25% utilization of the total Li deposit (8 mAh cm^{-2}). Insets show magnified voltage profiles in selected cycles.

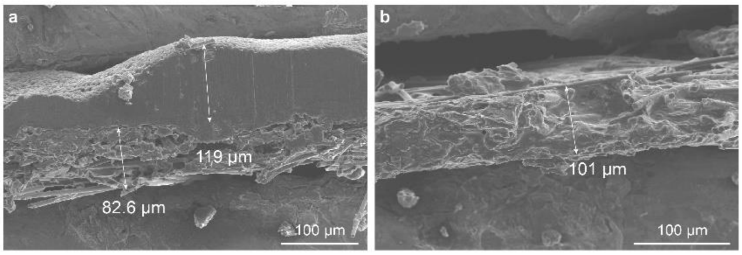

Supplementary Figure 18. a, b) SEM images of a) p-CP , b) d-CP after Li deposition at 0.5 mA cm^{-2} and 16 mAh cm^{-2}

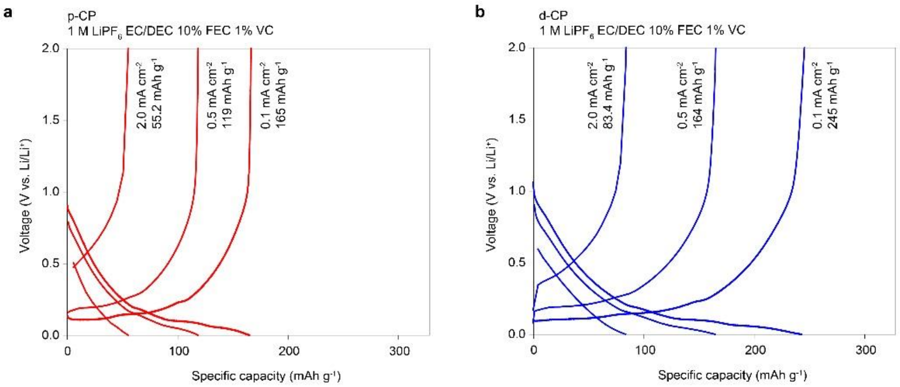

Supplementary Figure 19. Li intercalation and deintercalation potential profiles at various current densities for a, p-CP **b,** d-CP

According to the half-cell tests (Supplementary Fig. 19) and areal loading (Supplementary Table 3), the areal intercalation capacity is 0.908, 0.654 and 0.304 mAh cm^{-2} for p-CP and 0.956, 0.651 and 0.322 mAh cm^{-2} for d-CP at 0.1, 0.5, and 2.0 mA cm^{-2}, respectively. The Li plating capacity for 2.0 mA cm^{-2} and 4 mAh cm^{-2} in anode-free cell operation is 3.70 mAh cm^{-2} for p-CP and 3.68 mAh cm^{-2} for d-CP.

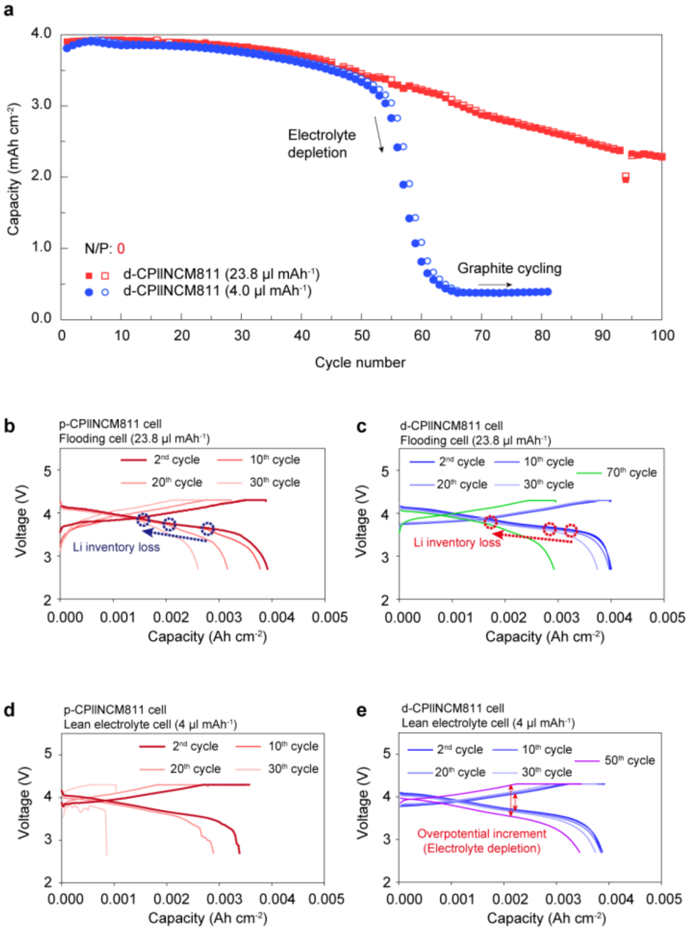

Supplementary Figure 20. NMC811 anode-free LMB cell test. a) Comparison of capacity fading between the flooded and lean electrolyte d-CP||NCM cell. **b-e)** Voltage profiles at various cycles for b, c) the flooded electrolyte cells and d, e) lean electrolyte cells (b, d) p-CP||NMC811, and c, e) d-CP||NMC811).

The comparison of cycling stability between the flooding and lean electrolyte d-CP||NCM811 cells shows a faster capacity fade from a certain cycle number for the lean electrolyte cell (Supplementary Fig. 20a). In the charging/discharging curves for the flooded electrolyte cells (Supplementary Fig. 20b, c), the discharge voltage dropped sharply at a certain capacity (marked by the dashed line circles), resulting in a decrease of capacity with cycle. The appearance of this point is associated with the depletion of the ‘lithium inventory’ in the cell. The voltage profiles for the lean electrolyte cells feature an increase of cell overvoltage with cycling due to an increased resistance with the depletion of the electrolyte (Supplementary Fig. 20d, e). This explains the faster capacity fade for the lean electrolyte cell (Supplementary Fig. 20a).

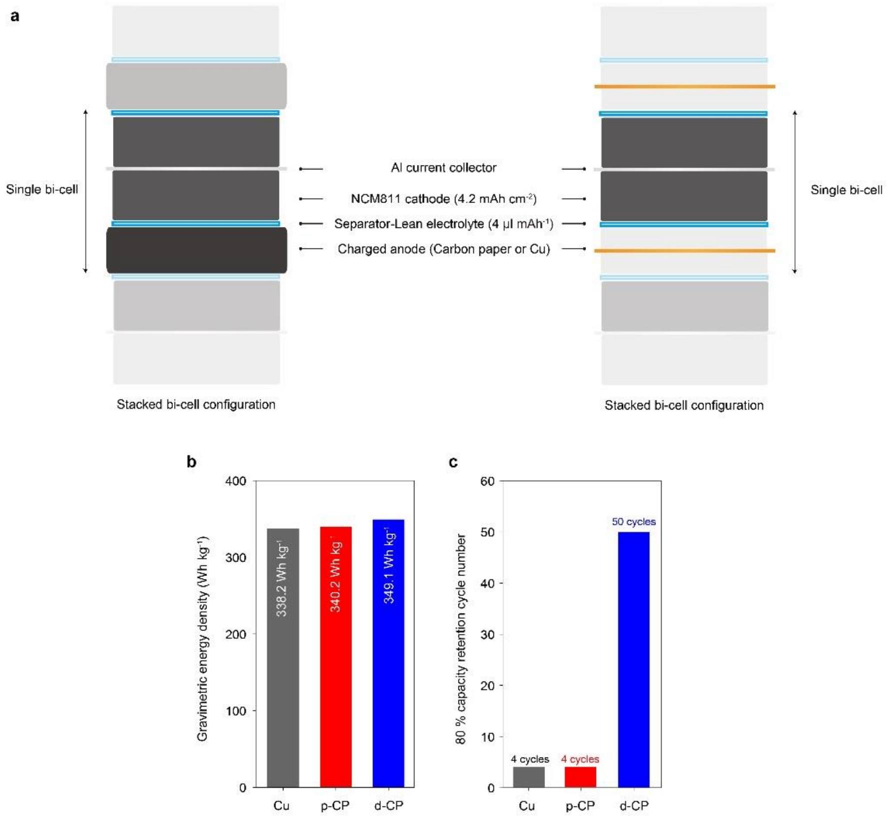

Supplementary Figure 21. Gravimetric energy density and cycle life of anode-free LMB cells. a) Schematic illustration of a stacked anode-free LMB. **b)** Estimated gravimetric energy densities of the anode-free LMB with different current collectors. c) Number of cycles at 80% capacity retention for the anode-free LMBs with different current collectors.

The gravimetric energy densities of a stacked anode-free LMB with different current collector were calculated based on 0.1 C charge/discharge voltage profile (Fig. 7a). In this calculation, the single bi-cell constitutes one sheet of double-side coated NCM cathode, one sheet of fully charged anode (current collector + deposited Li), two sheet of separator and electrolyte[24]. Here, in the case of Cu or p-CP, the thickness of the negative electrode increases significantly as Li deposition, but in the case of Cu, it is assumed that 5 μm per 1 mAh cm^{-2}, and in the case of p-CP, lithium is well deposited into the pore.

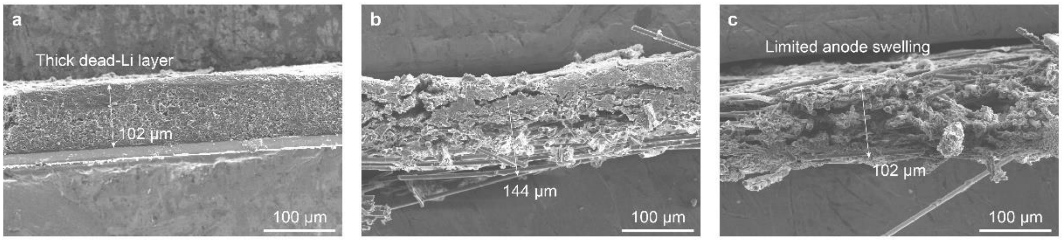

Supplementary Fig. 22. a-c, SEM images fully discharged state of anode current collectors after 10 cycles of 5

mAh cm^{-2} Li plating/stripping at 2 mA cm^{-2} for Cu (a), p-CP (b) and d-CP (c). Electrolyte: 1 M $LiPF_6$ EC/DEC 1:1 v/v 10% FEC 1% VC

In the case of the Cu current collector, it can be seen that a porous layer of about 100 μm is formed in only 10 cycles. When a bi-cell structured cell is constructed, the thickness of the single stack will be increased by 200 μm (100 μm each side), resulting in almost twice the volumetric energy density reduction (thickness vary from 251 at μm fully charged state to 413 μm at fully discharged state after 10 cycles), resulting volumetric energy density of 790.8 Wh l^{-1}. On the other hand, in the case of d-CP, it was confirmed that the thickness of the current collector was almost constant even after cycling.

Supplementary Table 1. Chemical compositions of p-CP, ZIF-8@p-CP, and d-CP determined by the EDX elemental mappings.

	Weight %			
**Electrodes**	**Carbon**	**Oxygen**	**Nitrogen**	**Zinc**
**p-CP**	97.48	2.52	-	-
**ZIF-8@p-CP**	87.67	2.31	6.97	3.05
**d-CP**	96.92	3.08	0.00	0.00

Supplementary Table 2. Operation conditions, Coulombic efficiencies, and cycling stability of Li plating/stripping process for previously reported current collectors.

**Strategy**	**Condition**	**Electrolyte**	**Coulombic efficiency**	**Reference**
Ni@Li_2O-NW	1 mA cm^{-2}<br>2 mAh cm^{-2}	1M LiTFSI DOL/DME<br>1% $LiNO_3$	92.5%<br>(180 cycles)	15
g-C_3N_4@Ni	1 mA cm^{-2}<br>2 mAh cm^{-2}	1M LiTFSI DOL/DME<br>1% $LiNO_3$	97%<br>(140 cycles)	16
3D Cu@N-graphene	2 mA cm^{-2}<br>4 mAh cm^{-2}	1M LiTFSI DOL/DME<br>1% $LiNO_3$	97%<br>(70 cycles)	17
3D Cu-Zn	1 mA cm^{-2}<br>1 mAh cm^{-2}	1M LiTFSI DOL/DME<br>1% $LiNO_3$	98.3%<br>(160 cycles)	18
Hierarchical Cu@Sn	3 mA cm^{-2}<br>1 mAh cm^{-2}	1M LiTFSI DOL/DME<br>2% $LiNO_3$	96.9%<br>(130 cycles)	19
Al_2O_3-Ni-Au 3D gradients	0.5 mA cm^{-2}<br>2 mAh cm^{-2}	1M LiTFSI DOL/DME<br>1% $LiNO_3$	97%<br>(350 cycles)	20
N plasma-CuO@Cu	1 mA cm^{-2}<br>1 mAh cm^{-2}	1M LiTFSI DOL/DME<br>1% $LiNO_3$	98.7%<br>(500 cycles)	21
ZnO@hierarchical Carbon	1 mA cm^{-2}<br>1 mAh cm^{-2}	1M LiTFSI DOL/DME<br>0.1M $LiNO_3$	97.1%<br>(200 cycles)	22
Aligned CuO@Cu	1 mA cm^{-2}<br>1 mAh cm^{-2}	1M LiTFSI DOL/DME<br>1% $LiNO_3$	94%<br>(180 cycles)	23
**MV defective carbon current collector**<br>**(This work)**	**2 mA cm^{-2}**<br>**5 mAh cm^{-2}**	**1M $LiPF_6$ EC/DEC**<br>**10% FEC 1% VC**	**98.6%**<br>**(170 cycles)**	

Supplementary Table 3. Calculated electrode porosity and the maximum capacity of lithium metal storage in the pores of the electrodes.

Electrodes	Apparent density (g cm^{-3})	Carbon fiber density (g cm^{-3})	Porosity	Pore volume (cm^3 per 1 cm^{-2})	Maximum Li accommodation (mAh cm^{-2})
**p-CP**	0.55	2.26	0.757-	$7.57x10^{-3}$	15.6
**d-CP**	0.39	2.26	0.827	$8.27x10^{-3}$	17.1

Supplementary Table 4. Estimated gravimetric and volumetric energy densities of anode-free LMB cells with different current collector.

	**Cu**	**p-CP**	**d-CP**
Al current collector (13 µm)	3.51 mg cm^{-2}	3.51 mg cm^{-2}	3.51 mg cm^{-2}
Anode current collector	7.17 mg cm^{-2} (8 µm)	5.5 mg cm^{-2} (100 µm)	3.9 mg cm^{-2} (100 µm)
NMC811 active material (74 µm)	20.79 mg cm^{-2}	20.79 mg cm^{-2}	20.79 mg cm^{-2}
PP separator (20 µm)	1.95 mg cm^{-2}	1.95 mg cm^{-2}	1.95 mg cm^{-2}
Electrolyte	E/C=4.84 g Ah^{-1}	E/C=4.84 g Ah^{-1}	E/C=4.84 g Ah^{-1}
0.1 C discharge capacity	4.24 mAh cm^{-2}	4.14 mAh cm^{-2}	4.24 mAh cm^{-2}
Average voltage	3.860 V	3.844 V	3.850 V
Total stack energy	32.74 mWh cm^{-2}	31.83 mWh cm^{-2}	32.65 mWh cm^{-2}
Total stack weight	96.8 mg cm^{-2}	93.5 mg cm^{-2}	93.5 mg cm^{-2}
Total stack thickness (Charged)	251 µm (ideally)	301 µm (ideally)	301 µm
**Stack gravimetric energy density**	**338.2 Wh kg^{-1}**	**340.2 Wh kg^{-1}**	**349.1 Wh kg^{-1}**
**Stack volumetric energy density**	**1304.4 Wh l^{-1}**	**1057.1 Wh l^{-1}**	**1084.7 Wh l^{-1}**
**80% capacity retention cycle #**	**4 cycles**	**4 cycles**	**50 cycles**

References

1 Zhang, X., Xu, Z., Ge, Z., Ouyang, X. & Ji, W. Tunable molecular configuration for significant enhancement of two-photon absorption based on novel octupolar benzoimidazole derivatives. *J. Photochem. Photobiol. A* **290**, 22-30 (2014).

2 Aikebaier, F., Pertsova, A. & Canali, C. M. Effects of short-range electron-electron interactions in doped graphene. *Phys. Rev. B* **92**, 155420 (2015).

3 Fu, F. *et al.* Size-Controllable Synthesis of Zeolitic Imidazolate Framework/Carbon Nanotube Composites. *Crystals* **8**, 367 (2018).

4 Yang, C.-M. *et al.* Nanowindow-regulated specific capacitance of supercapacitor electrodes of single-wall carbon nanohorns. *J. Am. Chem. Soc.* **129**, 20-21 (2007).

5 Utsumi, S. *et al.* Opening mechanism of internal nanoporosity of single-wall carbon nanohorn. *J. Phys. Chem. B* **109**, 14319-14324 (2005).

6 Joho, F. *et al.* Relation between surface properties, pore structure and first-cycle charge loss of graphite as negative electrode in lithium-ion batteries. *J. Power Sources* **97**, 78-82 (2001).

7 Foss, C. E. L. *Thermal Stability and Electrochemical Performance of Graphite Anodes in Li-ion Batteries.* (2014).

8 Chun, S.-E., Choi, J. & Whitacre, J. Tailoring the porous texture of activated carbons by CO 2 reactivation to produce electrodes for organic electrolyte-based EDLCs. *Ionics* **24**, 2055-2061 (2018).

9 Luo, G., Li, D., Yuan, G. & Li, N. J. J. o. T. E. S. Potentiostatic Current Transient for Multiple Nucleation: A Limited-Diffusion Process Description. *J. Electrochem. Soc.* **165**, D147-D151 (2018).

10 Alemu, T., Assresahegn, B. D. & Soreta, T. R. J. P. E. A. Tuning the initial electronucleation mechanism of palladium on glassy carbon electrode. *Port. Electrochim. Acta* **32**, 21-33 (2014).

11 Okamoto, Y. J. T. J. o. P. C. C. Density functional theory calculations of lithium adsorption and insertion to defect-free and defective graphene. *J. Phys. Chem. C* **120**, 14009-14014 (2016).

12 Hou, T. Z. *et al.* Design principles for heteroatom-doped nanocarbon to achieve strong anchoring of polysulfides for lithium–sulfur batteries. *Small* **12**, 3283-3291 (2016).

13 Huang, J.-X., Csányi, G., Zhao, J.-B., Cheng, J. & Deringer, V. L. J. J. o. M. C. A. First-principles study of alkali-metal intercalation in disordered carbon anode materials. *J. Mater. Chem. A* **7**, 19070-19080 (2019).

14 Pande, V. & Viswanathan, V. Computational Screening of Current Collectors for Enabling Anode-Free Lithium Metal Batteries. ACS Energy Lett. **4**, 2952-2959 (2019).

15 Zou, P. C. *et al.* Ni@Li2O co-axial nanowire based reticular anode: Tuning electric field distribution for homogeneous lithium deposition. *Energy Storage Mater.* **18**, 155-164 (2019).

16 Zuo, T. T. *et al.* Graphitized Carbon Fibers as Multifunctional 3D Current Collectors for High Areal Capacity Li Anodes. *Adv. Mater.* **29** (2017).

17 Zhang, R. *et al.* N-Doped Graphene Modified 3D Porous Cu Current Collector toward Microscale Homogeneous Li Deposition for Li Metal Anodes. *Adv. Energy Mater.* **8**, 1800914 (2018).

18 Fan, H., Dong, Q., Gao, C., Hong, B. & Lai, Y. J. M. L. Powder-sintering derived 3D porous current collector for stable lithium metal anode. *Mater. Lett.* **234**, 69-73 (2019).

19 Luo, Z. *et al.* Dendrite-free lithium metal anode with lithiophilic interphase from hierarchical frameworks by tuned nucleation. *Energy Storage Mater.* **27**, 124-132 (2020).

20 Pu, J. *et al.* Conductivity and lithiophilicity gradients guide lithium deposition to mitigate short circuits. *Nat. Commun.* **10**, 1896 (2019).

21 Luan, J. *et al.* Plasma-Strengthened Lithiophilicity of Copper Oxide Nanosheet–Decorated Cu Foil for Stable Lithium Metal Anode. *Adv. Sci.* **6**, 1901433 (2019).

22 Jin, C. *et al.* 3D lithium metal embedded within lithiophilic porous matrix for stable lithium metal batteries. *Nano Energy* **37**, 177-186 (2017).

23 Zhang, C. *et al.* Vertically aligned lithiophilic CuO nanosheets on a Cu collector to stabilize lithium deposition for lithium metal batteries. *Adv. Energy Mater.* **8**, 1703404 (2018).

24 Louli, A. J. *et al.* Diagnosing and correcting anode-free cell failure via electrolyte and morphological analysis. *Nat. Energy* **5**, 693-702 (2020).
